# Transcriptional Regulation of Glucose Metabolism: The Emerging Role of the HMGA1 Chromatin Factor

**DOI:** 10.3389/fendo.2018.00357

**Published:** 2018-07-03

**Authors:** Eusebio Chiefari, Daniela P. Foti, Riccardo Sgarra, Silvia Pegoraro, Biagio Arcidiacono, Francesco S. Brunetti, Manfredi Greco, Guidalberto Manfioletti, Antonio Brunetti

**Affiliations:** ^1^Department of Health Sciences, University “Magna Græcia” of Catanzaro, Catanzaro, Italy; ^2^Department of Life Sciences, University of Trieste, Trieste, Italy; ^3^Department of Medical and Surgical Sciences, University “Magna Græcia” of Catanzaro, Catanzaro, Italy; ^4^Department of Clinical and Experimental Medicine, University “Magna Græcia” of Catanzaro, Catanzaro, Italy

**Keywords:** HMGA1, glucose homeostasis, insulin resistance, type 2 diabetes, glucose metabolism

## Abstract

HMGA1 (high mobility group A1) is a nonhistone architectural chromosomal protein that functions mainly as a dynamic regulator of chromatin structure and gene transcription. As such, HMGA1 is involved in a variety of fundamental cellular processes, including gene expression, epigenetic regulation, cell differentiation and proliferation, as well as DNA repair. In the last years, many reports have demonstrated a role of HMGA1 in the transcriptional regulation of several genes implicated in glucose homeostasis. Initially, it was proved that HMGA1 is essential for normal expression of the insulin receptor (INSR), a critical link in insulin action and glucose homeostasis. Later, it was demonstrated that HMGA1 is also a downstream nuclear target of the INSR signaling pathway, representing a novel mediator of insulin action and function at this level. Moreover, other observations have indicated the role of HMGA1 as a positive modulator of the Forkhead box protein O1 (FoxO1), a master regulatory factor for gluconeogenesis and glycogenolysis, as well as a positive regulator of the expression of insulin and of a series of circulating proteins that are involved in glucose counterregulation, such as the insulin growth factor binding protein 1 (IGFBP1), and the retinol binding protein 4 (RBP4). Thus, several lines of evidence underscore the importance of HMGA1 in the regulation of glucose production and disposal. Consistently, lack of HMGA1 causes insulin resistance and diabetes in humans and mice, while variations in the *HMGA1* gene are associated with the risk of type 2 diabetes and metabolic syndrome, two highly prevalent diseases that share insulin resistance as a common pathogenetic mechanism. This review intends to give an overview about our current knowledge on the role of HMGA1 in glucose metabolism. Although research in this field is ongoing, many aspects still remain elusive. Future directions to improve our insights into the pathophysiology of glucose homeostasis may include epigenetic studies and the use of “omics” strategies. We believe that a more comprehensive understanding of HMGA1 and its networks may reveal interesting molecular links between glucose metabolism and other biological processes, such as cell proliferation and differentiation.

## Introduction

Glucose homeostasis is essential for life, and its maintenance is ensured through evolutionarily conserved regulatory mechanisms, that implicate complex and fine-tuned interplays between a variety of organs, tissues, hormones, receptors, nutrients, sensors, enzymes, and other molecules that may act locally and systemically (1, 2). In a physiological setting, the earliest mechanisms regulating postprandial hyperglycemia involve: (i) the readily releasable pool of insulin granules; (ii) the membrane translocation of glucose transporters in insulin-target tissues; (iii) post-translational regulatory mechanisms, mostly based upon post-translational modifications (i.e., phosphorylation), which affect enzyme functions that are implicated in carbohydrate metabolism, glucose homeostasis and disposal. Instead, during fasting conditions, when blood glucose levels are low, glucagon secretion increases to activate glycogenolysis and gluconeogenesis, thereby promoting hepatic glucose production to maintain fasting euglycemia. On a longer time-scale, instead, other effective mechanisms take place, which entail the transcriptional activation of genes and gene networks that function to control glucose homeostasis. In this context, glucose and insulin may regulate several “metabolic” genes by modulating the activity of nuclear factors toward their target genes (3). For example, it has been shown that glucose influences *insulin* gene transcription by inducing the phosphorylation of the glucose-sensitive PDX-1 transcription factor in pancreatic beta cells (4), while insulin can inhibit genes by triggering the phosphorylation of the forkhead box protein O1 (FoxO1), and its consequent relocation from the nucleus to the cytoplasm (5, 6). However, although in the last decades many studies have contributed to a better understanding of the transcriptional regulation of glucose metabolism, the role and interplay of several nuclear transcription factors in this scenario need further elucidation.

The high mobility group A1 (HMGA1) protein (also formerly known as HMGI/Y) is an architectural transcription factor involved in global chromatin remodeling. By interacting with both DNA and transcription factors, it regulates many fundamental biological processes, ranging from embryonic development to cell proliferation and differentiation, apoptosis, senescence and repair of DNA (7–13). Before the last two decades, HMGA1 has been mainly studied for its role in oncology, and to a lesser extent, in inflammation (9, 10, 14). Later, as part of investigations aimed at understanding the molecular basis of regulation of insulin receptor (*INSR*) gene expression, HMGA1 has emerged as a crucial factor in the transcriptional regulation of the *INSR* gene, and other genes relevant to glucose metabolism (15–18). Within this metabolic context, novel HMGA1 molecular partners have been identified, and their functional interplay investigated, while, in the meantime, *HMGA1* gene variants have been identified as reliably linked to both type 2 diabetes mellitus, and the metabolic syndrome (19–21).

The purpose of this review is to summarize current information on the structural and functional characteristics of HMGA1, and its role in the transcriptional regulation of the metabolic genes so far investigated. In this scenario, HMGA1 emerges as a crucial factor in the regulation of glucose production and disposal. Also, a recently recognized role of the *HMGA1* gene locus as a favored locus for susceptibility to insulin resistance and metabolic diseases is discussed, while future research directions are proposed to gain further insights into the links between HMGA1 and the pathophysiology of glucose metabolism and homeostasis.

## General characteristics of HMGA1

### Gene structure, transcriptional regulation, and protein synthesis

**Figure 1:**
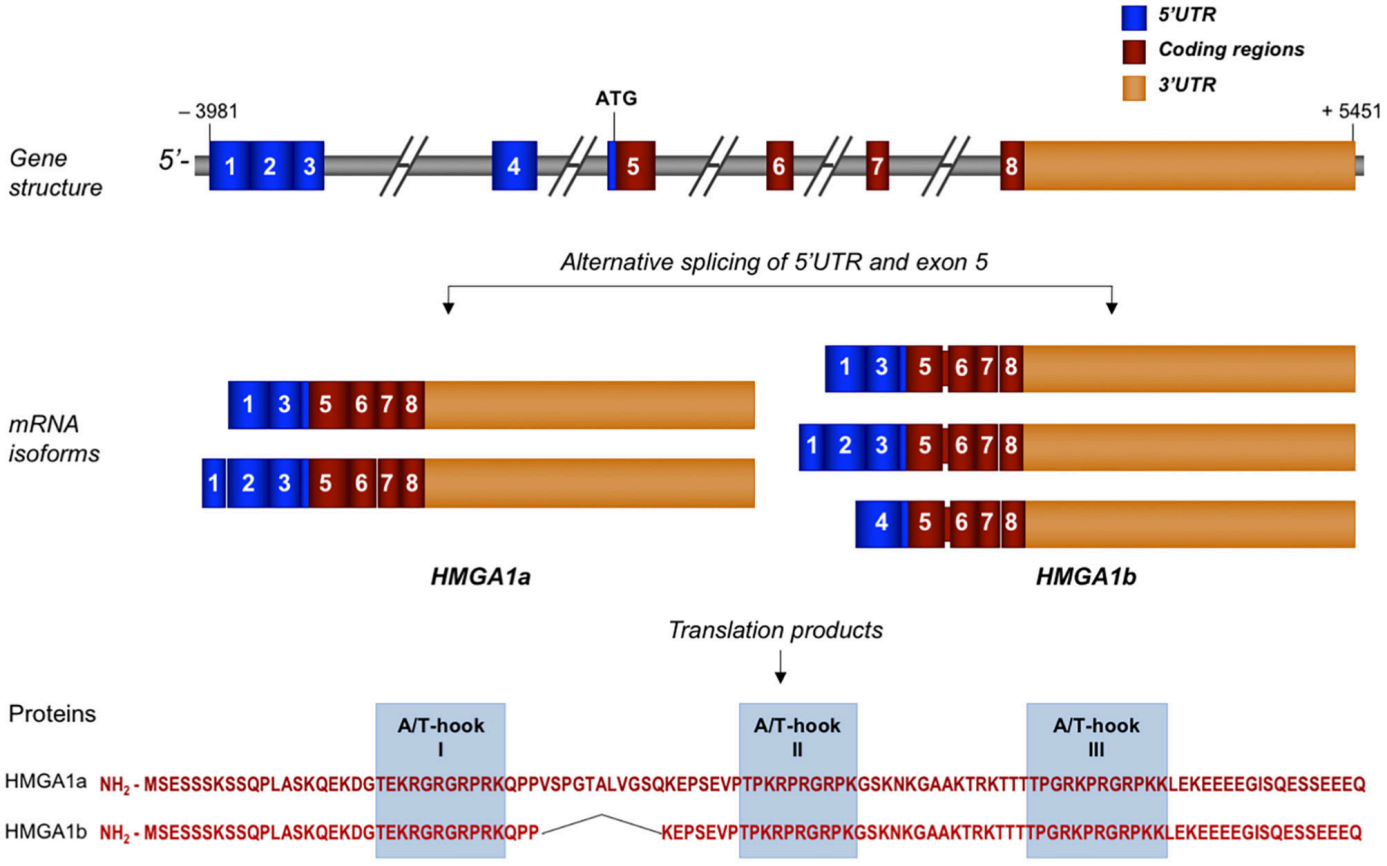
Schematic representation of human HMGA1 gene, transcripts, and protein isoforms. Exons are indicated by colored, numbered boxes. Main mRNA isoforms for both HMGA1a and HMGA1b are illustrated. Aminoacid sequence with the three functional AT-hook domains of both isoform proteins are reported.

In humans, the *HMGA1* gene is located on chromosome 6p21 (NC_000006.12), and it is well conserved among species (22, 23). Cloning and characterization of this gene reveal a very complex genomic organization, which includes a 5′ untranslated region (UTR) that undergoes alternative splicing, a coding region distributed on four exons, and a large 3′UTR (Figure 1). As for other genes, it is plausible that the size of the 5′UTR may influence the stability and translation efficacy of HMGA1 transcripts (24). The regulatory region of the *HMGA1* gene is highly GC-rich as a whole and lacks TATA and CAAT box sequences; also, it includes at least two transcription start sites with different promoter/enhancer regions (22, 25, 26), as it occurs for genes that are regulated without the preferential selection of any specific start site. However, a privileged utilization of start site 2 has been demonstrated in certain cell types and under certain experimental conditions (26), thus indicating a tight gene regulation that results in the transcription of specific mRNAs in response to different stimuli. In addition, it has been reported that the human *HMGA1* gene displays a basal transcriptional activity mainly controlled by the specificity protein 1 (Sp1) and the activator protein 1 (AP1) transcription factors, both of which stimulate *HMGA1* gene expression from the transcription start site 1 and the transcription start site 2, respectively (27).

A functional c-Myc-Max consensus DNA binding site was identified in the *HMGA1* promoter and, consistent with this, the oncoprotein c-Myc and its protein partner Max bind to this site and activate *HMGA1* gene transcription (28). Also, the HMGA1 promoter is activated by the transforming growth factor-β1 (TGF-β1) (29). Recently, we identified an octamer motif ATGCAAAT at the beginning of exon 1, where the octamer transcription factors Oct-1 and Oct-2 exert a differential regulation of *HMGA1* gene transcription, and demonstrated that, by binding its own promoter, HMGA1 can contribute to its transactivation by Oct-2 (30), thus supporting a previous observation about the role of HMGA1 in an auto-regulatory circuit (31). More recently, it has been reported that G9a, an activator of gene transcription and a histone methyltransferase, positively regulates the expression of the *HMGA1* gene in hepatic cells (32).

The complexity of the *HMGA1* gene structure and transcriptional regulation results in the generation of a series of mRNA isoforms that are largely derived from an extensive alternative splicing in the 5′UTR (Figure 1). Instead, the coding sequence undergoes only one differential splicing that produces the two protein isoforms, HMGA1a, consisting of 107 aminoacids, and HMGA1b, which lacks 11 aminoacids at the end of exon 5 (33) (Figure 1). Both protein isoforms contain three AT-hook DNA binding domains, a protein-protein interaction domain, which overlaps with the second AT-hook and includes the aminoacid sequence up to the third AT- hook, and a highly negative and constitutively phosphorylated C-terminal tail (11). Each DNA binding domain includes the core peptide motif Pro-Arg-Gly-Arg-Pro (P-R-G-R-P) (Figure 1), through which HMGA1 preferentially interacts with the minor groove of AT-rich DNA sequences (34, 35). Although all three AT-hook motifs synergize during target recognition, the first two AT-hooks contribute to the majority of HMGA1 affinity for DNA (36). The two different HMGA1 isoforms may have different biological functions, as indicated by studies in MCF-7 breast epithelial cells, where HMGA1b forced expression confers a more aggressive neoplastic phenotype than HMGA1a (37). However, in the context of other cell lines or of other biological processes, including metabolism, more investigations are needed to deepen this issue.

Being among the most abundant non-histone, chromatin-associated protein, HMGA1 has been shown to cooperate with other nuclear proteins, including the chaperone nucleophosmin (38), and to play a role in the chromatin organization by an interplay with histones (39, 40). Interactions of HMGA1 with transcription factors will be later discussed in the “DNA, protein and RNA interactions” paragraph.

### Post-transcriptional regulation

The functional activity of HMGA1 relies on a complex and fine regulation of its own expression. This includes a series of regulatory elements that act within the 3′UTR HMGA1 mRNA (41). In addition, recent studies indicate that many microRNAs could bind the HMGA1 3′UTR mRNA, causing its degradation or inhibiting its translation (42). Down-regulation of some of these microRNAs—miR15, miR-16, miR26, miR-196a-2, and let-7—have been described to cause increased levels of HMGA1 in pituitary adenomas (42). Interestingly, some of the same miRNAs involved in tumorigenesis also play a role in metabolism. For example, miR-26a has been shown to target key regulators of insulin signaling and glucose metabolism in the liver, while its impairment is associated with hepatic oncogenesis and metabolic disorders (43).

Another peculiar mechanism of HMGA1 post-transcriptional regulation refers to the role of processed pseudogenes as potential regulators of mRNA stability/degradation (44). Processed pseudogenes are non-functional copies of normal genes generated by a process of mRNA retrotransposition. Compared with homologous normal genes, they lack introns and contain single nucleotide substitutions, deletions, insertions, and residues of poly (A) tails (45, 46). Human genome includes thousands of pseudogenes, accumulated during evolution (45, 46). However, although our actual knowledge about the real biological role of pseudogenes is still limited, increasing evidences exist, supporting a functional significance for these macromolecules (47). So far, eight HMGA1 pseudogenes have been described (48). Some of them act on the stability of HMGA1 mRNA or prevent miRNAs from targeting *HMGA1* mRNA, thereby behaving as competing endogenous RNAs (ceRNAs). The RNA encoded by one of them, the *HMGA1-p* pseudogene, by effectively competing for the trans-acting cytoplasmic protein αCP1, accelerates the degradation of mRNA from the homolog normal gene, thereby reducing the longevity of HMGA1 mRNA transcript (44). Some pseudogenes display aminoacid sobstitutions at the level of specific aminoacid residues that in the native HMGA1 are subjected to post-translational modifications involved in the modulation of HMGA1's activities. An intriguing possibility is that, if expressed, these proteins could compete with the native HMGA1, escaping the modulatory effects of these post-translational modifications that strongly impact on HMGA1 ability for chromatin remodeling and protein-protein interactions (48).

### Post-translational modifications (PTMs)

A variety of extracellular and intracellular signals can induce different PTMs on HMGA1 protein, which influence its ability to interact with either DNA or proteins, thereby affecting HMGA1 nuclear function (8, 49). PTMs include phosphorylation, methylation, and acetylation (8, 12, 49, 50). Generally, increased level of HMGA1 phosphorylation reduces DNA-binding affinity and transcriptional activation, and this status correlates with an elevated residence time of the HMGA1a isoform in the repressed inactive heterochromatin, rather than in the active euchromatin (35). In detail, phosphorylation can affect different serine (Ser) and threonine (Thr) residues. The phosphorylation at Thr-52 and Thr-77 by the cell-cycle dependent kinase cdc2 results in decreased binding of HMGA1a to DNA (51, 52). The same effect is produced following the phosphorylation of HMGA1a at Ser-43 and Ser-63 by the protein kinase C (PKC) pathway (53). The acidic C-terminal tail of HMGA1 is constitutively phosphorylated. At this level, the protein kinase CK2 catalyzes the phosphorylation of Ser-98, Ser-101, and Ser-102 of HMGA1a (54), while it has been demonstrated that phosphorylation of the C-terminal tail has structural consequences on HMGA1 compactness (55). HMGA1 is also susceptible to acetylation on several lysine residues (49). It has been reported that acetylation by the histone acetyltransferases CBP (CREB-binding protein) and p300/CBP associated factor (PCAF)/GCN5 have a role in the kinetics of enhanceosomes assembly/disassembly. Acetylation at Lys-64, by CBP, destabilizes the enhanceosome formation on the human interferon beta (*IFN-beta*) gene, leading to transcriptional inhibition of this gene; on the contrary, PCAF-induced acetylation at Lys-70 increased the transcription of the *IFN-beta* gene through enhanceosome stabilization (56). HMGA1 is also methylated at several residues located exclusively within the AT-hook motifs. Although the significance of this type of PTM is still largely unknown, methylation at the AT-hook motifs indicate a potential role for methylation in regulating HMGA1-DNA binding activity (12, 49, 57–59).

### Tissue expression of HMGA1

HMGA1 is highly expressed during embryogenesis, suggesting its critical role during the embryonic development (60). It is also highly expressed in adult stem cells, including intestinal and hematopoietic stem cells (61). The important role of HMGA1 at these levels is supported by phenotypic studies in *Hmga1-*knockout mice, indicating that mice lacking HMGA1 develop cardiomyopathy, aberrant hematopoiesis, and defective pancreatic beta cell development (19, 62). Also, HMGA1 plays a role in adipogenesis, and myogenesis, although its levels decrease before terminal cell differentiation (63, 64). Vice versa, overexpression of HMGA1 is found in a wide range of human cancers, including prostate, thyroid, colon, breast, lung, bladder, pancreas, stomach, kidney, uterus, and hepatocellular carcinomas, as well as non-melanoma skin cancers, and hematopoietic malignancies (10, 14, 65, 66). In some of these cancers, HMGA1 expression strongly correlates with an advanced stage, the metastatic potential and reduced survival. Poorer prognosis is due to the fact that HMGA1 promotes the transcription of many genes involved in tumor growth, invasion, migration, neoangiogenesis, epithelial-mesenchymal transition and cancer metastasis (8, 10, 67–71). Nevertheless, it has been also reported that HMGA1 can have anti-oncogenic effects, depending on the cellular context (72). This bivalent function proves the relevance of HMGA1 in both physiological and pathological conditions and explains the reason why HMGA1 requires a fine-tuned spatio-temporal expression and activity modulation.

### DNA, protein and RNA interactions

**Figure 2:**
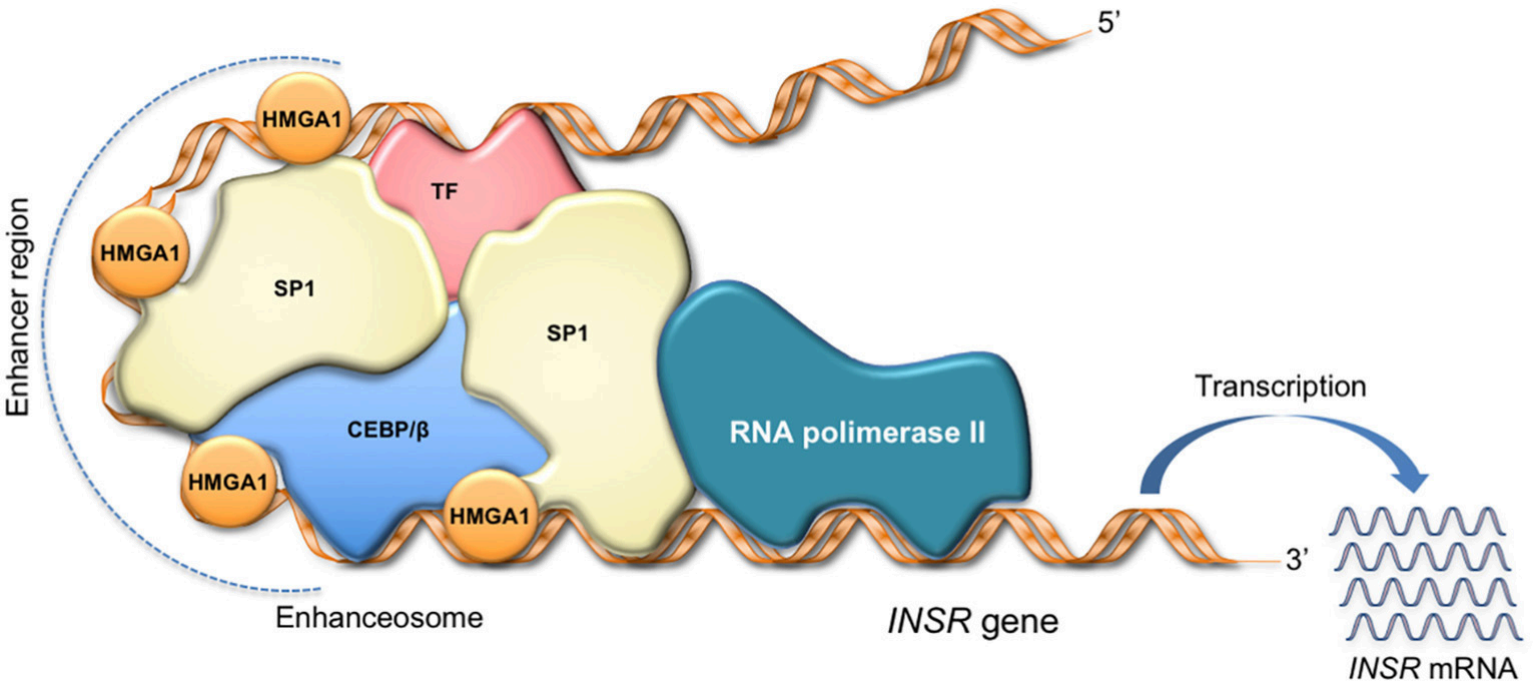
HMGA1 as facilitator of enhanceosome formation. HMGA1 binds to the enhancer region through its DNA-binding domain, while interacting with other transcription factors in the promoter, forming a multiprotein-DNA complex that enhances gene transcription. The scheme refers to activation of the *INSR* gene.

HMGA1 regulates cell cycle-related chromosomal changes, DNA replication and repair, and molecular chaperoning (11, 38, 73). Also, by inducing a more open chromatin state, HMGA1 assists gene transcription (8, 74). By itself, HMGA1 has no intrinsic transcriptional activity; rather, it can participate in the transactivation of gene promoters through mechanisms that facilitate the assembly and stability of stereospecific DNA-protein complexes, termed “enhanceosomes,” that drive gene transcription (Figure 2) (75–77). HMGA1 performs this task by interacting with a large variety of activating or inhibiting transcription factors and orchestrating their assembly on promoter regions. The enhanceosome model provides one of the best-understood examples of how HMGA1 can interact with other transcription factors, leading to a highly specific activation of gene transcription in higher eukaryotes (76, 77). In this sense, enhanceosome formation over the *IFN-beta* promoter (75, 77) or the *INSR* promoter (15) is of paradigmatic importance. In relation to protein-protein interactions, the HMGA1 network has been reported to be very wide (78, 79), as HMGA1 has been shown to physically and functionally interact with many ubiquitous and tissue-specific transcription factors. Although the repertoire is far for being complete, the interplays of HMGA1 with the transcription factors p53, NF-κB and ATF-2/c-Jun, C/EBP beta and Sp1, as well as PDX-1 and MafA have been studied in depth, being crucial, for example, in the transcriptional regulation of *Bcl 2* (80), *IFN-beta* (75), *INSR* (15), and *insulin* (18) genes, respectively. Besides C/EBPbeta and PDX-1, among the nuclear factors known to be involved in glucose metabolism, HNF-1alpha has been shown to bind and cooperate with HMGA1 in the regulation of the *IGFBP1* and *IGFBP3* promoters (16), whereas the peroxisome proliferator-activated receptor-gamma (PPARgamma), which reduces *INSR* gene transcription, failed to show any direct interaction with HMGA1 in this context (81). The potential interplay of HMGA1 with other nuclear metabolic sensors, such as ChREBP, SREBP1-c, FoxO family members, and PGC-1, instead, has not yet been investigated.

Emerging lines of evidence indicate that HMGA1 interacts also with different RNAs. The first evidence supporting a specific RNA affinity of HMGA1 has been reported in studies pointing to a role of HMGA1 during the exon skipping of presenilin-2 pre-mRNA, which resulted in the production of a deleterious protein isoform causing sporadic Alzheimer's disease (82). More recently, a new interaction has been reported between HMGA1 and the nuclear non-coding 7SK RNA (83), a factor which negatively affects Polymerase II transcription elongation and influences HMGA1 biological functions by competing for the first AT-hook binding with DNA (84). Also, through the first AT-hook, HMGA1 has been shown to interact with the origin recognition complex, thus playing a key role in DNA replication (85).

## HMGA1 and glucose homeostasis

### HMGA1 in the regulation of the INSR gene

The peptide hormone insulin exerts its biological effects by binding to the INSR, a specific tyrosine kinase receptor glycoprotein located in the plasma membrane of insulin target cells. As a key regulator of insulin action, many studies have explored the *INSR* gene (86, 87). Nuclear binding proteins that recognized the *INSR* promoter were initially identified during muscle and adipose cell differentiation in the context of two AT-rich sequences of the regulatory region of the *INSR* gene (88). Using conventional chromatographic purification methods, combined with electrophoretic mobility shift assays and immunoblots, these proteins were identified as HMGA1, while reporter gene analysis findings showed that HMGA1 is required for proper transcription of the *INSR* gene (89). Further studies demonstrated that transcriptional activation of the human *INSR* gene required the assembly of a transcriptionally active multiprotein-DNA complex, including the ubiquitously expressed transcription factor Sp1 and C*/*EBPbeta, in addition to HMGA1 (15). As HMGA1 physically interacts with these proteins and facilitates their binding to DNA, functional integrity of this protein-DNA complex is necessary for full transactivation of the *INSR* gene promoter by Sp1 and C*/*EBPbeta (15). In support of the role of HMGA1 in *INSR* gene transcription, *in vitro* investigations in beta-pancreatic cells demonstrated that sustained hyperglycemia impaired HMGA1 expression, a condition affecting INSR content in beta cells and, thus, insulin secretion (90).

These observations, which were based mainly on *in vitro* analyses, were substantiated by studies *in vivo*, in *Hmga1-*knockout mice, in which a marked decrease in *INSR* gene and protein expression was observed in the major targets of insulin action, contributing to a phenotype characteristic of human type 2 diabetes (19). Studies in patients with low INSR as a consequence of defects in HMGA1 will be discussed in the “HMGA1 in insulin resistant diseases” section, while discrepancies between human and mouse phenotypes will be discussed later.

Subsequent investigations revealed that other transcription factors, such as the activating protein 2 (AP2) and PPARgamma, can influence *INSR* gene transcription in a variety of cell types (81, 91), while studies in cultured myocytes aimed at deciphering the mechanisms by which free fatty acid (FFA) contribute to the development of insulin resistance and type 2 diabetes, showed that FFA can impair INSR expression and insulin signaling and sensitivity by affecting HMGA1 (92–95). In particular, FFA induce phosphorylation and nuclear translocation of the protein kinase C epsilon type (PKCepsilon). In the nucleus, PKCepsilon phosphorylates HMGA1 and downregulates its expression by deactivating the transcription factor Sp1, thereby attenuating *INSR* gene expression by direct and indirect mechanisms, which in turn compromise insulin action and sensitivity (92–95). Furthermore, recent studies addressed the mechanisms linking the downregulation of the histone methyltransferase G9a/EHMT2 with insulin resistance in murine models and in cultured human hepatic cells. G9a/EHMT2 upregulates HMGA1 and G9a knockdown hepatic cells showed reduced INSRs, whose expression was restored by overexpressing HMGA1 (32). Importantly, restoration of G9a levels in db/db mice improved hepatic insulin signaling and ameliorated hyperglycemia and hyperinsulinemia at least in part by upregulating HMGA1 (32). Altogether, these findings clearly indicate that HMGA1 is a crucial component of the insulin signaling pathway, and plays an important role in *INSR* gene expression in insulin target tissues.

### HMGA1 in the transcription of the insulin (INS) gene

A direct role of HMGA1 in insulin production and pancreatic islet development and beta cell function has been postulated starting from the observation that, compared to wild-type littermates, *Hmga1-*knockout mice showed decreased insulin secretion and reduced beta cell mass (19). On the other hand, a functional interplay between HMGA1 and the homeodomain transcription factor PDX-1 (a key regulator of pancreatic islet development and beta cell function) has been shown previously in the context of the *INS* gene and other pancreatic islet-specific genes (96). The possibility for HMGA1 to play a role also in this context, was substantiated by the fact that binding of PDX-1 to the *INS* gene promoter was reduced in *Hmga1-*knockout mice (19). Also, a protein-protein interplay between PDX-1, the neurogenic differentiation 1 (NeuroD1), and HMGA1 had been previously described at the level of the rat insulin mini-enhancer element E2A3/4 (96). Subsequent studies added more details in our understanding of the *INS* gene regulation. In the insulin-secreting beta-cell line INS-1, as demonstrated by chromatin immunoprecipitation experiments, glucose stimulated binding of HMGA1 to the *INS* promoter, resulting in a significant increase in insulin production and secretion (18). Coherently, when INS-1 cells were treated with HMGA1 siRNA, a significant reduction in glucose-induced insulin secretion was observed, thereby confirming the importance of HMGA1 in this scenario (18). Even in the absence of HMGA1-DNA binding sites on the *INS* gene promoter, the assembly of a transcriptionally active multiprotein-DNA complex involving HMGA1, PDX-1 and the transcription factor MafA, was required for proper transcription of both human and mouse *INS* genes (18). In line with this observation, the deficit in HMGA1 compromised binding of PDX-1 and MafA to the *INS* promoter, thereby imparing *INS* gene transcription and glucose-induced insulin secretion (18). However, given that substantial interspecies differences exist in pancreatic islet development and function (19, 97, 98), any parallelism between human and mouse at this level must be considered carefully and further details on this should be provided. For example, based on our recent observations highlighting a novel relationship between HMGA1 and FoxO1 (99), further investigation in this field could deliver deeper information on the possibility that an interplay among HMGA1 and FoxO1 can be a component of this regulation, as an overarching role of FoxO1 in pancreatic beta cell function has been already outlined (6, 100–103).

### HMGA1 as a downstream target of the INSR signaling pathway

Besides being required for both *insulin* and *INSR* gene transcription, HMGA1 plays an important role in the regulation of the insulin signaling cascade (98). The gluconeogenic genes phosphoenolpyruvate carboxykinase (*PEPCK*) and glucose-6-phosphatase (*G6Pase*), as well as the *IGFBP1* gene (which plays a glucose counterregulatory role by preventing the potential hypoglycemic effects of IGF1) are known to be inhibited by insulin (for example, after a meal). As reported *in vitro*, in hepatic cells, insulin, via the phosphatidylinositol 3-kinase (PI-3K)/Akt pathway, and the Akt/protein kinase CK2 signaling, exerts its transcriptional repression of these genes by inducing HMGA1 phosphorylation (104). In fact, by triggering the phosphorylation of HMGA1 at the level of the three serine residues, Ser-98, Ser-101, and Ser-102, insulin promotes the detachment of HMGA1 from promoter target genes and its corresponding nuclear localization in the inactive heterochromatin. Thus, HMGA1 acts as a downstream modulator of insulin action, and is an important key player in insulin and nutritionally-regulated transcription of genes involved in glucose metabolism and homeostasis. Also, as phosphorylation/dephosphorylation of HMGA1 can act as a molecular switch for deactivating or activating INSR protein expression during fed and fasting conditions, respectively (104), HMGA1 can function as a key feedback regulator of insulin signaling during the fasting and refeeding periods.

Given that the role of the transcription factor FoxO1 in the control of gluconeogenesis is well established (6, 105), as for the regulation of pancreatic beta cell function, a cross-talk between HMGA1 and FoxO1 can be hypothesized also in this case and investigated in future studies.

### HMGA1 and insulin-independent metabolic signaling

Data from the *Hmga1*-knockout mouse model evidenced a complex metabolic phenotype, in which peripheral insulin hypersensitivity paradoxically coexisted with a condition of impaired glucose tolerance and overt diabetes (19), thus supporting the existence of alternative insulin signaling pathways ensuring peripheral glucose utilization and disposal by insulin-independent mechanisms. Coexistence of insulin hypersensitivity in peripheral tissues with insulin resistance has been observed before in liver of *ob/ob* mice (106) and in *Cdk4* knockout mice with defects in pancreatic beta cell development and insulin secretion (107). The possibility that the activation of insulin-independent mechanisms aimed at ameliorating glucose disposal under disadvantageous metabolic conditions, like those affecting *Hmga1*-knockout animals, is underlined by the identification of novel biochemical pathways involving the cAMP-HMGA1-RBP4 system (17, 108) and the HMGA1-IGF1/IGFBP system (16), whose activation may play a role in glucose homeostasis in both rodents and humans. Further studies *in vitro* confirmed that HMGA1 has a role in the activation of both *IGFBP1* and *IGFBP3* gene transcription (16, 109). Therefore, it is plausible that under physiological circumstances (e.g., during fasting), in which HMGA1 increases (17, 108), the increment of both IGFBP1 and IGFBP3 helps in limiting IGF1 bioavailability, thereby preventing peripheral glucose uptake by insulin-independent mechanisms.

### Role of HMGA1 during fasting

The counter-regulatory hormone glucagon, which acts in opposition to insulin, binds its cognate G-protein coupled receptor on liver cell membrane and stimulates the transmembrane adenylyl cyclase to produce cyclic AMP (cAMP) as second messenger. This, in turn, leads to the activation of protein kinase A (PKA), which, among many other proteins, phosphorylates the Cyclic AMP Responsive Elements Binding Protein (CREB) transcription factor (110, 111). The final event is the assembly of a functional transcriptional machinery on the promoter regions of gluconeogenic genes (112). Some observations in cultured hepatic cells indicate that cAMP also increases HMGA1 protein expression (17, 108). Consistently, Hmga1 RNA levels were significantly increased in liver of mice following systemic administration of glucagon.

**Figure 3:**
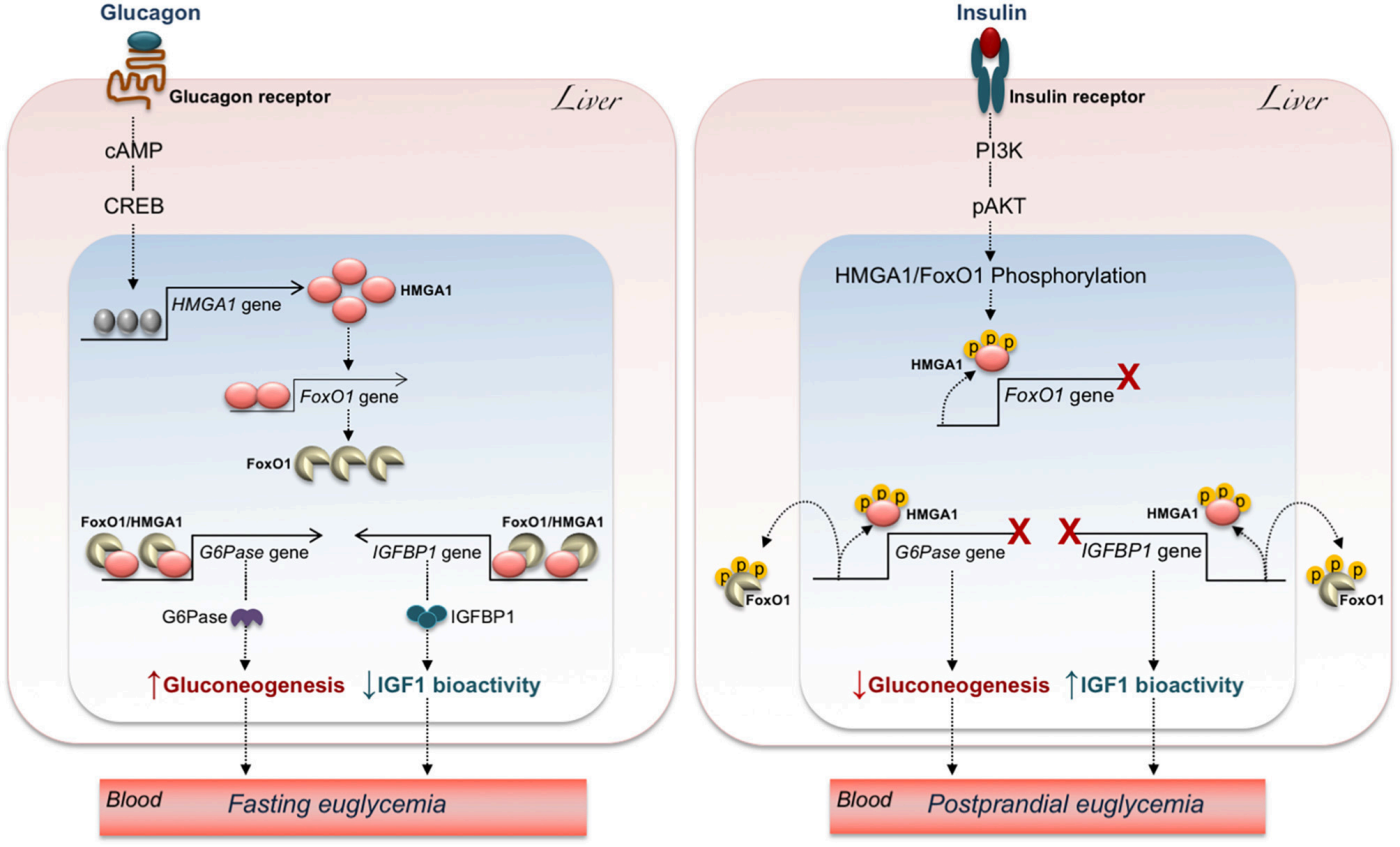
Hypothetical mechanisms underlying the effects of HMGA1/FoxO1 on glucose homeostasis. The increase of glucagon during fasting **(Left)** turns on the cAMP-PKA-CREB pathway, allowing *HMGA1* gene activation and protein expression. In turn, HMGA1 activates the *FoxO1* gene and promotes transactivation of *G6Pase* and *IGFBP1* promoters by FoxO1, thereby maintaining fasting euglycemia through elevation of hepatic gluconeogenesis and attenuation of IGF1 bioactivity. Under feeding conditions **(Right)**, binding of insulin to its receptor initiates a series of events culminating in the sequential phosphorylation (p) of HMGA1 and FoxO1, which reduces *FoxO1* gene expression, promotes the detachment of FoxO1 from *G6Pase* and *IGFBP1* gene promoters, and leads to FoxO1 nuclear exclusion, thereby ensuring postprandial euglycemia through inhibition of hepatic gluconeogenesis and augmentation of IGF1 bioactivity.

In agreement with the observations mentioned above, upregulation of FoxO1 expression via the glucagon-cAMP-PKA signaling has been reported in liver of fasting mice to maintain fasting euglycemia (113). Thus, upregulation of HMGA1 during fasting (when glucagon peaks) may contribute to the mechanisms necessary to prevent hypoglycemia, through activation of *FoxO1* (99) and gluconeogenic gene expression. The opposite occurs after a meal, when insulin peaks, and glucagon declines (Figure 3). In this metabolic scenario, inactivation of HMGA1 by insulin-induced HMGA1 phosphorylation, by causing the detachment of FoxO1 from DNA and its nuclear exclusion, inhibits gluconeogenesis and contributes to restoration of postprandial euglycemia (Figure 3).

Kidney exerts an important role in gluconeogenesis, being responsible of approximately 15% of glucose production (1). In a recent paper, after 3-day fasting or restriction diet in mice, renal gene expression, assayed by microarray, demonstrated, among other transcription factors, an increment in HMGA1 expression (114). These findings are coherent with previous findings in the liver, in which an effect of HMGA1 on gluconeogenic genes has been described (104).

Another glucose metabolism-related gene, which has been shown to be regulated by HMGA1, is the one encoding for the retinol binding protein 4 (RBP-4). RBP-4 is mostly produced by the liver, although adipose tissue also contributes, and plays a role in systemic insulin resistance. RBP-4 expression in fat and its levels in blood inversely correlated with the adipose-specific glucose transporter GLUT-4 in obesity and type 2 diabetes (115). *In vitro* studies with human HepG2 and murine Hepa 1 hepatoma cells have demonstrated that HMGA1 binds to and increases transcription of the *RBP-4* gene promoter both in basal and in cAMP-induced conditions (17, 108), while *in vivo*, in whole mice, injection of glucagon, by inducing increased intracellular cAMP, activates both HMGA1 and RBP-4 expression in liver and fat. Consequently, under physiological circumstances, this loop has an important relapse in conditions of low glucose availability, in which intracellular cAMP increases. In this scenario, HMGA1, by inducing FoxO1-dependent gluconeogenic genes, and by upregulating RBP-4, helps providing glucose to tissues/organs with high energy demands, such as the brain. Interestingly, the brain-type GLUT-3 facilitative glucose transporter has also been shown to be transcriptionally regulated by HMGA1 (116), thereby supporting further the relevance of this factor in multiple settings of energy demand.

### HMGA1 in adipose and muscle cell differentiation and function

Both muscle and fat play relevant roles in maintaining euglycemia. In this regard, previous studies from our group demonstrated that INSR expression is reduced in muscle and adipose tissues from both *Hmga1*-knockout mice and in individuals with reduced levels of HMGA1 (19, 44).

The physiological role of HMGA1 in adipogenesis has been investigated *in vitro* and *in vivo* (63, 117), and a critical role of HMGA1 in adipocytic cell growth and differentiation has been demonstrated in murine 3T3-L1 adipocytes (117). Also, HMGA1 may exert a negative role in adipose cell growth by balancing the effects of the cognate HMGA2 protein, another member of the HMGA family (118). Indeed, transgenic mice, overexpressing HMGA1 in both white and brown adipose tissues, showed reduced fat mass and impaired adipogenesis with respect to wild-type mice (63), and were protected against high-fat diet induced obesity and systemic insulin resistance, thus supporting the role of HMGA1 in the maintenance of glucose homeostasis.

In addition to RBP-4, whose regulation has been discussed, other adipokines have been demonstrated to be under the control of HMGA1. In 3T3-L1 adipocytes, visfatin, an insulin-mimetic factor, is transcriptionally regulated by HMGA1 in cooperation with the hypoxia-inducible factor 1, HIF-1 (119), whereas leptin, an adipokine involved in glucose and fatty acid metabolism, is regulated by HMGA1 through a non-canonical mechanism that spares HMGA1 direct binding to DNA and requires the physical interaction and functional cooperation of HMGA1 with the nuclear factor C/EBPbeta (117).

Several reports have also indicated that HMGA1 plays a role in muscle tissue, and HMGA1 is present in mouse C2C12 cultured muscle cells, in which HMGA1 overexpression increases cell proliferation and prevents myotube formation (64). Downregulation of HMGA1 is an early and necessary step for the progression of the myogenic program. In this regard, it has been reported that miR-195/497, by binding the HMGA1 3′UTR, reduces HMGA1 protein abundance in C2C12 cells, thus promoting muscle cell differentiation (120).

The Lin28/let-7 pathway, whose implication in cancer is well known (121), displays also a role in the regulation of glucose metabolism. In fact, mice overexpressing Lin28a and Lin28b show an insulin-sensitized state, with protection against high-fat diet induced diabetes (122). In contrast, muscle-specific loss of Lin28a and overexpression of let-7 resulted in insulin resistance and impaired glucose tolerance (122). As Lin28a directly promotes HMGA1 translation (123), it has been postulated that in muscle-specific Lin28a knockout mice, insulin resistance is, at least in part, due to reduced HMGA1 levels and consequently impaired INSR expression (122). If confirmed in further studies, the relationship between HMGA1 and the Lin28/let-7 pathway may indicate another molecular mechanism for the involvement of HMGA1 in mammalian glucose metabolism.

## HMGA1 in insulin resistant diseases

### Syndromes of severe insulin resistance

Insulin resistance, defined as a subnormal biological response to the glucose-lowering effect of insulin, is a characteristic of many common disorders, including type 2 diabetes, the metabolic syndrome, fatty liver disease, and obesity (124–126). However, severe forms of insulin resistance may occur as uncommon syndromes, either congenital or acquired, in patients with impaired INSR signaling or lipodistrophy (127, 128). Congenital disorders include the Type A syndrome of insulin resistance, the Rabson-Mendenhall syndrome, leprechaunism, and some syndromes of generalized or partial lipodystrophy. Type A syndrome is an autosomal dominant disorder characterized by the triad of hyperinsulinemia, acanthosis nigricans, and ovarian hyperandrogenism (127–129). Hyperglycemia is not always present at diagnosis. Female patients appear lean and without lipodystrophy, even if a variant of this syndrome has been reported in obese women (130, 131). Male patients may initially exhibit acanthosis nigricans and hypoglycemia, while overt diabetes may not occur until the fourth decade or later (128). In some cases, the syndrome is caused by heterozygous mutations affecting the tyrosine kinase domain of the INSR; however, only 10–25% of female with Type A syndrome have mutations in the *INSR* gene (132).

As a step toward understanding the molecular basis of regulation of the *INSR* gene, a nuclear binding protein that specifically interacted with, and activated the *INSR* gene promoter, was identified previously, during muscle and adipose cell differentiation (88). Later, this DNA binding protein was identified as HMGA1, and its expression was markedly reduced in two unrelated patients with either the Type A syndrome or the common form of type 2 diabetes, in whom cell surface INSRs were decreased and *INSR* gene transcription was impaired despite the fact that the *INSR* genes were normal, thus indicating defects in *INSR* gene regulation (15, 89, 133). Subsequent investigations in both these patients allowed the identification of a novel genetic variant, c.^*^369del, in the 3′ non-coding region of HMGA1 mRNA, which resulted in a decreased mRNA half-life and reduced HMGA1 protein levels. In other two patients (mother and daughter) with the type A syndrome of insulin resistance, a hemizygous deletion of the *HMGA1* gene was also identified (19). Restoration of HMGA1 protein expression in these subjects' cells enhanced *INSR* gene transcription and restored cell-surface INSR protein expression, thus confirming that defects in HMGA1, by decreasing INSR protein production may indeed induce severe insulin resistance (19).

The mechanistic linkage between HMGA1, insulin resistance and certain less common forms of type 2 diabetes has been further supported by a study in two diabetic patients, in whom aberrant expression of a pseudogene for HMGA1, *HMGA1-p*, caused destabilization of HMGA1 mRNA with consequent loss of INSR and generation of insulin resistance (44).

These findings demonstrate, therefore, that HMGAl is necessary for proper expression of the INSR. Further, they provide evidence for recognizing “HMGA1opathy” as a novel diabetic subphenotype (134).

### Type 2 diabetes

In its common form, type 2 diabetes is a heterogeneous complex disease in which concomitant insulin resistance and beta-cell dysfunction lead to hyperglycemia (135, 136). From a pathogenetic point of view, both predisposing genetic factors and precipitating environmental factors contribute importantly to the development of the disease (135, 136). So far, about 100 gene variants have been associated with an increased risk for type 2 diabetes (137). Most of these variants are presumed to negatively affect pancreatic beta-cell function and insulin secretion, while some of them appear to impact peripheral insulin sensitivity, thereby impairing tissue glucose uptake (138).

As it concerns HMGA1, on the basis of its involvement in insulin resistance, a role for this nuclear factor in type 2 diabetes has also been postulated and studies in this direction have been performed by us and others (20, 139–141). In particular, by sequencing the entire *HMGA1* gene in a large number of diabetic patients and healthy controls, four variants of the *HMGA1* gene were identified by us in approximately 10% of diabetics (20). In circulating monocytes and cultured lymphoblasts from diabetic patients carrying these variants, HMGA1 and INSR expressions were markedly decreased and these defects were corrected by transfecting HMGA1 cDNA (20). The most frequent *HMGA1* rs139876191 variant (previously named rs146052672), was significantly higher in type 2 diabetic patients from three populations of white European ancestry: Italian, American and French populations (20). Although not replicated in a heterogeneous French population (139), the rs139876191 variant was later associated with type 2 diabetes among Chinese (140) and Americans of Hispanic ancestry (141), thus providing evidence for the implication of the *HMGA1* gene locus as one conferring a cross-race risk for the development of type 2 diabetes. More recently, the credibility of an association between the *HMGA1* rs139876191 variant and type 2 diabetes was confirmed also in a transethnic meta-analysis that included all available published articles examining this association in different populations (142). Like other polymorphisms located at the intron/exon boundaries, functional analysis of the *HMGA1* rs139876191 revealed that this variant is functional and exhibits a dominant negative effect (143).

### Metabolic syndrome

The metabolic syndrome is a common multicomponent disorder, which is associated with increased risk for type 2 diabetes, cardiovascular disease (CVD), and nonalcoholic fatty liver disease (144, 145). As insulin resistance plays a pivotal role in the pathophysiology of metabolic syndrome (125, 146), the impact of HMGA1 has been investigated in two large Italian and Turkish populations, both affected by metabolic syndrome (21). Findings indicated that the *HMGA1* rs139876191 variant was significantly associated with metabolic syndrome in both populations, in which this association occurred independently of type 2 diabetes, thus lending credence to the hypothesis that this variant may independently associate with other insulin resistance-related traits. Consistent with this assumption, a strong association of the rs139876191 variant with certain metabolic syndrome-related traits (i.e., high fasting plasma glucose, high body mass index, low HDL-Cholesterol, reduced insulin sensitivity) was observed in affected individuals of European and Hispanic-American ancestry, further supporting the notion that defects that negatively affect HMGA1 can play a role in the pathogenesis of metabolic syndrome and other insulin-resistance related conditions (21, 141). Interestingly, as CVD is a major risk for both type 2 diabetes and the metabolic syndrome, the association of *HMGA1* rs139876191 variant with acute myocardial infarction, independently from diabetes and other cardiovascular risk factors, has been reported previously (147, 148), suggesting that *HMGA1* may also represent a novel genetic marker of cardiovascular risk.

### Discrepancy between human and mouse phenotypes with HMGA1 loss-of-function

An important issue that deserves to be discussed is to which extent *Hmga1*-knockout mice reflect findings in humans. Although in the broader context of glucose metabolism similarities between the two species are well known (i.e., in both species, insulin and glucagon represent key effectors in the control of glucose homeostasis), differences are likewise described in relation to pancreas development and, in particular, to the late stages of beta cell differentiation and susceptibility to pancreatic beta-cell injury (97, 149, 150). At a molecular level, previous known beta cell species-specificities in ion channel components and membrane transporters, as well as in insulin secretion, have been recently further enriched by data from transcriptome profiles in single human and murine beta cells (150, 151), while evidence of heterogeneity of pancreatic beta cells has been proved to occur in both humans and mice (152). However, interspecies differences do not exclude that in some instances, like in the case of lack of the *KCNJ11* gene, the mouse phenotype well recapitulates human neonatal diabetes (153).

Focusing on HMGA1 loss-of-function, three biochemical and metabolic conditions are common to humans and mice: reduced insulin receptor expression, impaired insulin signaling, and insulin resistant diabetes. Instead, insulin levels in humans (hyperinsulinemic) and mice (hypoinsulinemic) are clearly discrepant (19). In fact, in *Hmga1*-knockout mice, both beta cell mass and insulin secretion are impaired. Differences in pancreatic islet ontogenesis and differentiation, as well as differences in nongenetic environmental elements and susceptibility to genetic modifiers, have been postulated to explain these dissimilarities (19). On the other hand, *Hmga1*-knockout mice have proved to be insulin hypersensitive, despite the deficit in INSRs. This apparent paradox suggests the presence of adaptive, compensatory mechanisms, in mice, that include the already described cAMP-HMGA1-RBP4 pathway and the IGFBP-1/IGF1 system. Although the latter has proved to be more effective to reduce glycemia in mice than in humans (154), the importance of these systems in both species still deserves further investigations. As an example, recent findings obtained in genetically engineered mice with a specific deletion of the *RBP4* gene in the liver, indicating that circulating RBP4 derives mainly from hepatocytes (155), need to be confirmed in humans.

## Conclusions

At present, HMGA1 is known to be involved in multiple biological processes. Based on the above-mentioned findings, among the many tasks that HMGA1 can perform, there is its role in the transcriptional regulation of gene and gene networks involved in INSR signaling and glucose metabolism. In this review, we provided an overview of the major contributions that have been made in this area over the last years. Overall, the data obtained so far well support the role of HMGA1 in the regulation of genes implicated in the maintenance of glucose homeostasis and metabolic control, providing new insight into the regulation of glucose metabolism and disposal. Clinically, the importance of *HMGA1* gene variability in glucose metabolism is emphasized in a wide range of clinical conditions ranging from rare insulin resistance syndromes to type 2 diabetes and the metabolic syndrome. Besides, being a multifunctional protein, HMGA1 may constitute a molecular link between metabolism and other distinct biological processes, including cell proliferation, and differentiation, viability, autophagy, cell cycle, apoptosis, that need to be sustained by cell energy.

New insights may come from epigenetic studies, including miRNAs, whose common role in both malignancy and metabolism is recently emerging. On the other hand, disentangling the pleiotropic nature of HMGA1 by the identification of distinct molecular partners and networks uniquely implicated in metabolism, still represents a big challenge. A contribution could come from studies on the relationship between HMGA1 and the yet unexplored nuclear metabolic sensors.

Apart from the intrinsic biological and clinical interest of these findings, a deeper understanding of the mechanisms that regulate glucose metabolism in health and disease is of importance for the development of more effective therapies. To fill the gap of our knowledge in this regard, future directions based on the omics-related technologies, combined with bioinformatic tools, can help identify novel proteins and their networks, as well as genes and gene products regulated by, or interacting with HMGA1.

To the best of our knowledge, this is the first review article exclusively dedicated to the role of HMGA1 in this context, and we hope that it will serve as a quickly accessible reference in this important clinical field.

## Author contributions

EC and DF prepared the first draft of the manuscript. RS, SP, MG, and GM contributed to critical revision of the manuscript. BA and FB were involved in the literature search. AB critically revised and edited the final version of the manuscript.

### Conflict of interest statement

The authors declare that the research was conducted in the absence of any commercial or financial relationships that could be construed as a potential conflict of interest. The handling editor is currently co-organizing a Research Topic with one of the authors AB, and confirms the absence of any other collaboration.
